# Comprehensive Genetic Analysis of a Hungarian Amyotrophic Lateral Sclerosis Cohort

**DOI:** 10.3389/fgene.2019.00732

**Published:** 2019-08-16

**Authors:** Kornélia Tripolszki, Piyush Gampawar, Helena Schmidt, Zsófia F. Nagy, Dóra Nagy, Péter Klivényi, József I. Engelhardt, Márta Széll

**Affiliations:** ^1^Department of Medical Genetics, University of Szeged, Szeged, Hungary; ^2^Research Unit for Genetic Epidemiology, Gottfried Schatz Research Center, Molecular Biology and Biochemistry, Medical University of Graz, Graz, Austria; ^3^Department of Neurology, University of Szeged, Szeged, Hungary

**Keywords:** amyotrophic lateral sclerosis, oligogenic inheritance, next-generation sequencing, mutation screening, *C9orf72* repeat expansion, genetic heterogeneity

## Abstract

Amyotrophic lateral sclerosis (ALS) is a progressive neurodegenerative disease characterized by the degeneration of motor neurons. Genetic factors play a key role in ALS, and identifying variants that contribute to ALS susceptibility is an important step toward understanding the etiology of the disease. The frequency of protein altering variants in ALS patients has been extensively investigated in populations of different ethnic origin. To further delineate the genetic architecture of the Hungarian ALS patients, we aimed to detect potentially damaging variants in major and minor ALS genes and in genes related to other neurogenetic disorders. A combination of repeat-sizing of *C9orf72* and next-generation sequencing (NGS) was used to comprehensively assess genetic variations in 107 Hungarian patients with ALS. Variants in major ALS genes were detected in 36.45% of patients. As a result of repeat sizing, pathogenic repeat expansions in the *C9orf72* gene were detected in 10 patients (9.3%). According to the NGS results, the most frequently mutated genes were *NEK1* (5.6%), *NEFH*, *SQSTM1* (3.7%), *KIF5A*, *SPG11* (2.8%), *ALS2*, *CCNF*, *FUS*, *MATR3*, *TBK1*, and *UBQLN2* (1.9%). Furthermore, potentially pathogenic variants were found in *GRN* and *SIGMAR1* genes in single patients. Additional 33 novel or rare known variants were detected in minor ALS genes, as well as 48 variants in genes previously linked to other neurogenetic disorders. The latter finding supports the hypothesis that common pathways in different neurodegenerative diseases may contribute to the development of ALS. While the disease-causing role of several variants identified in this study has previously been established, other variants may show reduced penetrance or may be rare benign variants. Our findings highlight the necessity for large-scale multicenter studies on ALS patients to gain a more accurate view of the genetic pattern of ALS.

## Introduction

Amyotrophic lateral sclerosis (ALS) is a fatal neurodegenerative disease characterized by the degeneration of upper and lower motor neurons (UMN and LMN, respectively) in the motor cortex, brain stem, and spinal cord, with a life expectancy of 3–5 years from symptom onset (Peters et al., 2015). Approximately 5–10% of all cases show a family history of ALS (fALS), whereas the remaining 90–95% seem to occur sporadically (sALS, Renton et al., 2014); nevertheless, fALS and sALS cases are indistinguishable regarding their clinical features. The genetic background of ALS is complex: more than 30 major genes have been associated with the disease, and more than 100 additional genes have been associated with disease risk (Amyotrophic Lateral Sclerosis Online Database, Abel et al., 2012). Variants of these genes have been implicated in several pathological mechanisms of ALS, including protein homeostasis, RNA metabolism, endosomal and vesicular transport, DNA repair, excitotoxicity, mitochondrial dysfunction, autophagy, nucleocytoplasmic transport, oligodendrocyte degeneration, axonal transport, and neuroinflammation (Hardiman et al., 2017; van Damme et al., 2017). The significance of trans-ethnic study design for human genetics is broadly documented (Morris, 2011; Asimit et al., 2016). Variants in the same genes are thought to contribute to the genetic etiology of both fALS and sALS (Renton et al., 2014). According to twin studies, the estimated heritability of sALS is about 60% (Al-Chalabi et al., 2010). Pathogenic variants have been described in 40–80% of fALS cases and in 5–15% of sALS patients (van Damme, 2018).

We have previously reported the prevalence and clinical characteristics of Hungarian patients with variants in the *SOD1*, *SETX*, and *C9orf72* genes (Tripolszki et al., 2017a; Tripolszki et al., 2017b). The aim of the present study was to investigate the variants in the set of genes that have been associated with ALS so far, using next-generation sequencing (NGS) and repeat sizing of the *C9orf72* gene in a cohort of 107 clinically well-characterized Hungarian patients.

## Patients and Methods

### Patients

Patient recruitment was performed by senior neurologists at the Department of Neurology, University of Szeged. One hundred four patients were of Hungarian and three patients were of Romanian origin (total n = 107). Patients were unrelated to other enrolled patients and met the revised El Escorial and Awaji-shima criteria for ALS (de Carvalho and Swash, 2009; Ludolph et al., 2015). Regarding the family history of the patients, only one patient (#122u) showed a positive family history, and two patients (#99u and #93u) had relatives with uncertain diagnosis of ALS. Four patients (#91u, #90u, #87u, and #56r) had first- or second-degree relatives with other neurodegenerative diseases (Parkinson’s or Alzheimer’s disease). The samples of 37 patients (without pathogenic variants) from the previously studied cohort (Tripolszki et al., 2017a) were also used in this analysis. The whole cohort was prescreened by Sanger sequencing for two major ALS genes (*SOD1* and *TARDBP*), and no disease-causing variants were detected.

Because Hungarian population-specific databases have not been established yet, variants originating from whole-exome sequencing (WES) data of other studies were used as control in-house database. This in-house database included variants of 184 individuals (without any neurological diseases) of Hungarian (n = 133, mean age: 51 years) or Austrian (n = 51, mean age: 67.5 years) origin. To assess the frequency of the detected R261H *NEK1* variant in the Hungarian general population, an additional 186 samples from healthy individuals (mean age: 67 years) were used.

### Methods

#### DNA Extraction

Genomic DNA was isolated from whole EDTA-containing venous blood using the DNeasy^®^ Blood & Tissue Kit (QIAGEN, Gödöllő, Hungary).

#### C9orf72 Repeat Expansion Detection

A two-step protocol was applied for the detection of the GGGGCC repeat expansion (RE) in the *C9orf72* gene. A sizing PCR was performed to determine the number of hexanucleotide repeats in the normal range as described previously (Akimoto et al., 2014). Normal repeat length was defined as ≤28 repeats. Samples revealing a single peak product were further analyzed by long-read PCR using the AmplideX PCR/CE *C9orf72* (RUO) Assay (Asuragen, Inc.) as previously described (Suh et al., 2018). Amplidex PCR technique uses gene-specific and repeat-specific primers and provides an accurate capillary electrophoresis sizing of alleles up to 145 GGGGCC repeats and identifies the presence of expanded alleles over 145 repeats.

#### Next-Generation Sequencing

Patient DNA was target-enriched using a custom design SureSelect panel containing 247 genes (see **Supplementary Table 1** for gene lists) in 86 patients and Human All exon V6 kit in 21 patients (Agilent Technologies, Santa Clara, CA, USA), according to the manufacturer’s recommendations. Sequencing was performed on Illumina NextSeq 500 sequencer (Illumina Inc., San Diego, CA, USA). As a result of sequencing, the mean on-target coverage was 189× in case of the panel data and 71× per base in case of whole exome data with an average percentage of targets covered greater or equal to 10× of 96% and 90%, respectively. Data analysis was performed according to the best practices to identify single-nucleotide variants and small insertions/deletions. Paired-end reads were aligned to the Human Reference Genome (UCSC Genome Browser build hg19) using the Burrows–Wheeler Aligner (BWA). Duplicates were marked using the Picard software package. Genome Analysis Toolkit (GATK) was used for variant calling (BaseSpace BWA Enrichment Workflow v2.1.1. with BWA 0.7.7-isis-1.0.0, Picard: 1.79 and GATK v1.6-23-gf0210b3), and variants tagged “PASS” by GATK were used for downstream analysis and annotated using the ANNOVAR software tool (version 2017 July 17, Wang et al., 2010). In case of whole exome data, the variant files were parsed for genetic variants in genes of the custom design SureSelect panel (247 genes, **Supplementary Table 1**). Raw reads of potentially relevant variants were manually checked using the Integrative Genomics Viewer (Robinson et al., 2011; Thorvaldsdóttir et al., 2013).

#### Variant Filtering

Calls per sample with a read depth of <10 or an allele balance of <0.3, intronic and synonymous variants, and variants with a population frequency higher than 0.1% in either the ExAC Browser V0.3.1 (http://exac.broadinstitute.org) or 1000 Genomes Database (www.1000genomes.org/) were excluded from further analysis. Because the *SOD1* D91A variant is the most common known pathogenic variant seen in the Amyotrophic Lateral Sclerosis Online Database (ALSoD) with minor allele frequency (MAF) of 0.001, we used it as a criterion for filtering out variants with higher MAF. Other population databases were also used for additional variant information: Kaviar (version 2015 09 23; Glusman et al., 2011), dbSNP 138 (Sherry et al., 2002), and gnomAD (Lek et al., 2016). The combination of eight variant prioritization tools available from the dbNSFP database v3.0 (MetaSVM, M*etaL*R, DANN, PROVEAN, SIFT, Polyphen2, MutationTaster, MutationAssessor) was used to predict the effect of each variant on the corresponding protein (Liu et al., 2016). Variants identified in our patients were cross-checked in ALSoD (Abel et al., 2012), ALS Data Browser (ALSdb, http://alsdb.org) containing variants from 3,239 ALS cases and 11,808 controls (version v3 updated on Dec 03 2018), and ClinVar (Jan 30, 2017) databases, as well as in case reports in the literature. Variants that were found in our control in-house database—except for the R261H *NEK1* variant that was characterized by reduced penetrance—were excluded from further analysis. The detected variants were classified in accordance with the guideline of the American College of Medical Genetics and Genomics (ACMG, Richards et al., 2015). All genetic changes with a read depth <25 were validated by Sanger sequencing.

#### Gene Sets of Custom Design Panel

Based on the ALSoD and literature (Abel et al., 2012; Garton et al., 2017; Krüger et al., 2016), two gene sets containing all major and minor genes involved in ALS-associated pathways were generated: Set 1, categorized as major ALS genes, contained 30 genes that fulfilled the criteria for causation, and Set 2, contained 101 risk or candidate genes (ALSoD). A third extended gene set contained 116 genes related to other neurodegenerative and neuromuscular disorders (such as hereditary spastic paraplegia, spinal muscular atrophy, distal hereditary motor neuropathy, variants of Charcot–Marie–Tooth disease, distal myopathy, etc.) that may have genetic or symptomatic link to ALS (based on GeneReviews, Online Mendelian Inheritance in Man; Abel et al., 2012; Krüger et al., 2016; Garton et al., 2017; **Supplementary Table 1**).

#### Statistical Analysis

Statistical analysis of the R261H *NEK1* variant in patients and healthy individuals was carried out according to the guidelines of case–control allelic association study design (Lewis, 2002). All statistical analyses were performed using RStudio version 1.0.153 (RStudio Team, 2015). Frequencies were compared using *X* *^2^* statistics (p < 0.05).

## Results

A total of 107 ALS patients (62 females, 45 males; mean age at disease onset: 60 years; age range: 30–79 years) were analyzed in this study. All patients presented UMN and LMN signs, and 69% also presented bulbar signs.

Pathogenic repeat expansions in the *C9orf72* gene were detected in 10 patients (9.3%, mean age of onset: 60.8 years, age range: 49–72 years). According to NGS data, 28 variants were detected in 14 major ALS genes that passed the filtering criteria and assessment in Integrative Genome Viewer. Furthermore, we identified 33 variants in 26 minor ALS genes (**Supplementary Table 2**) and 48 variants in 31 genes associated with other neurodegenerative or neuromuscular diseases (**Supplementary Table 3**). No patients were identified as being homozygous for any of the studied variants.

### Genetic Variants Detected in Major ALS Genes

Combining NGS and repeat sizing, variants in major ALS genes were detected in 36.45% (39/107) of patients including patients with variants considered to be of uncertain significance (VUS). The detected variants have MAF below 0.001, with an exception for variants in NEK1, as these variants have a reduced penetrance (Nguyen et al., 2018). Based on ACMG variant classification, two of the 29 detected major ALS variants were categorized as pathogenic, four as likely pathogenic and the remaining 23 variants as VUS. The most common pathogenic genetic alteration was the *C9orf72* hexanucleotide RE, present in 9.3% of our patients, with all 10 patients carrying more than 145 GGGGCC repeats. Bulbar symptoms, primarily the alteration of the speech, was the initial sign in seven out of the 10 patients with *C9orf72* RE. None of these patients had dementia according to the information obtained from the relatives (in case the patients could not speak or move at the examination) or the results of the MMSE. Three patients had concomitant thyroid disease, and two had a disease of the cervical spine with myelopathy, which may have influenced the signs of ALS (**Table 1**).

**Table 1:** Patients carrying the C9orf72 repeat expansion.

Patient ID	Age at onset (age range group, years)	Duration before the 1^st^ exam	Early signs and symptoms	Signs at the 1^st^ exam	ALSFRS-R	MMSE	Other diseases	Other major ALS gene variant	Family history
#122u	70-75	1.4 y	dysarthria dysphagia	B, PB +++ LMN +++ UMN+	9/48	NA	stenosis of the cervical spinal canal and myelopathy	–	Younger sister and mother probably ALS
#108u	45-50	2 ys	dysarthria	B, PB +++ LMN +++ UMN ++	16/48	NA	–	SQSTM1, R393Q	no
#99u	55-60	8 months	dysarthria	B, PB +++ LMN ++ UMN +	36/48	30/30	Hypo-thyreoidism	NEK1, N250S	Mother questioned /no medical data/ Uncertain family history
#96u	70-75	3 months	Para-paresis	B, PB – LMN + UMN ++	37/48	28/30	–	–	no
#83r	50-55	1 y	dysarthria, dysphagia	B, PB +++ LMN + UMN++	37/48	28/30	Hashimoto thyroiditis	–	no
#85r	60-65	6 months	Peroneal palsy	B, PB – LMN + UMN+	41/48	30/30	Hashimoto thyroiditis CV-CVI-CVII disc herniation operated	–	no
#50u	55-60	1 y	dysarthria dysphagia	B, PB +++ LMN + UMN +	45/48	30/30	–	–	no
#63u	60-65	9 months	dysarthria dysphagia peroneal palsy	B, PB +++ LMN + UMN +	33/48	28/30	–	–	no
#88u	55-60	1 y	Dysarthria dysphagia	B, PB +++ LMN + UMN +	41/48	NA	CVI-VII Disc protrusion	–	no
#75r	65-70	5 months	Para-paresis	B, PB – LMN+++ UMN++	35/48	30/30	–	–	no

B, PB, Bulbar and pseudobulbar; UMN, upper motor neuron; LMN, lower motor neuron; ALSFRS-R, ALS Functional Rating Scale Revised; MMSE, Mini-Mental State Examination.

Apart from the repeat expansion, a rare missense variant of uncertain significance (R431Q) was also detected in the *C9orf72* gene. According to the NGS results, the most frequently mutated genes were *NEK1* (6/107, 5.6%), *NEFH*, *SQSTM1* (4/107, 3.7%), *KIF5A*, *SPG11* (3/107, 2.8%), *ALS2*, *CCNF*, *FUS*, *MATR3*, *TBK1*, and *UBQLN2* (2/107, 1.9%). Furthermore, potentially relevant variants were found in the *GRN* and *SIGMAR1* genes in single patients (**Table 2**). Because of the relatively high prevalence of the *NEK1* R261H variant in our patient cohort (5/107), we further evaluated 186 additional healthy controls (total 370) for this variant. R261H was identified in 5/107 (4.67%) patients and 4/370 (1.08%) controls, showing an enrichment in patients (MAF: 0.0234 vs 0.0054; p = 0.0162).

**Table 2:** Major ALS gene variants detected in the Hungarian ALS cohort.

Gene	Transcript	Nucleotide change	Amino acid change	PopMax MAF (ExAc)	dbSNP	ALSdb MAF ALS	ALSdb MAF Control	Pathogenicity (ACMG)	MetaSVM/ MetaLR/ PROVEAN/ SIFT/ PolyPhen2/ MutTast/ MutAs/ DANN	No patients	References	Patient ID
*ALS2*	NM_020919	c.G3529T	p.G1177X	0	rs386134180	0	0	Pathogenic	D/D/D/D/D/A/H/0.993	1	Sztriha et al., 2008	#62r
*ALS2*	NM_020919	c.G4496A	p.R1499H	2,49x10^–5^	rs566436589	0	0	VUS	T/T/D/D/P/D/L/1	1	–	#59r
*C9orf72*		GGGGCC Repeat expansion		0	–	0	0	Pathogenic	–	10	Renton et al., 2011; DeJesus-Hernandez et al., 2011	#99u, #108u, #75r, #50u, #63u, #88u, #96u, #85r, #83r, #122u
*C9orf72*	NM_001256054	c.G1292A	p.R431Q	5,83x10^–5^	–	0	0	VUS	T/T/N/T/D/D/L/0.999	1	–	#56u
*CCNF*	NM_001761	c.C1714T	p.R572W	4,14x10^–5^	rs199743115	0	4,24x10^–5^	VUS	T/T/D/D/D/D/M/0.999	1	–	#107u
*CCNF*	NM_001761	c.C316G	p.L106V	0	rs990719669	0	4,24x10^–5^	VUS	T/T/N/D/D/D/M/0.998	1	–	#85u
*FUS*	NM_001170937	c.A74G	p.Y25C	8,24x10^–6^	rs141516414	0	0	VUS	D/D/D/D/D/D/M/0.992	1	–	#87u
*FUS*	NM_001170937	c.C317T	p.P106L	2,47X10^–5^	rs374191107	0	4,24x10^-5^	VUS	T/T/N/T/B/D/M/0.997	1	Huey et al., 2012	#110u
*GRN*	NM_002087	c.T1003C	p.C335R	0	–	0	0	VUS	D/D/D/D/D/D/H/0.996	1	–	#106u
*KIF5A*	NM_004984	c.G2272A	p.E758K	5,54x10^–4^	rs140281678	1,5x10^–3^	8,90x10^–4^	VUS	T/T/N/D/B/D/N/0.998	2	–	#85u, #57r
*KIF5A*	NM_004984	c.G1735A	p.A579T	0	rs760135493	1,544x10^–4^	4,24x10^–5^	VUS	T/T/N/T/B/D/M/0.995	1	–	#83u
*MATR3*	NM_001194956	c.C31T	p.P11S	0	rs995345187	0	0	VUS	T/T/N/D/D/D/N/0.998	1	–	#58r
*MATR3*	NM_018834	c.G824A	p.S275N	0	–	0	0	VUS	T/T/N/T/B/D/N/0.99	1	–	#105u
*NEFH*	NM_021076	c.C1013T	p.T338I	0	rs774252076	0	0	VUS	D/D/D/D/D/D/M/0.997	2	–	#63r, #75u
*NEFH*	NM_021076	c.G443C	p.R148P	0	–	0	0	VUS	D/D/D/D/D/D/L/0.882	1	–	#69u
*NEFH*	NM_021076	c.C1514T	p.P505L	0	rs1414968372	1,609x10^–4^	0	VUS	T/T/D/D/B/N/L/0.843	1	–	#106u
*NEK1*	NM_001199397	c.G782A	p.R261H	3,73x10^–3^	rs200161705	6,6x10^–3^	3,30x10^–3^	VUS	T/T/D/D/P/D/M/0.999	5	Kenna et al., 2016; Brenner et al., 2016; Nguyen et al., 2018	#56r, #48r, #51, #90u, #93u
*NEK1*	NM_001199397	c.A749G	p.N250S	0	rs368762503	0	0	VUS	T/T/D/T/P/D/N/0.988	1	–	#99u
*SIGMAR1*	NM_001282205	c.T125G	p.I42R	0	rs1206984068	0	0	VUS	T/T/D/D/D/D/M/0.987	1	–	#73u
*SPG11*	NM_025137	c.G6101A	p.R2034Q	0	rs750101301	0	0	VUS	T/T/N/T/B/D/L/0.998	1	–	#64r
*SPG11*	NM_025137	c.C6352G	p.L2118V	8,72x10^–6^	rs766851227	0	4,25x10^–5^	VUS	D/D/N/D/D/D/M/0.998	1	–	#71u
*SPG11*	NM_025137	c.G6009T	p.E2003D	0	–	0	0	VUS	T/T/N/T/B/D/M/0.969	1	–	#104u
*SQSTM1*	NM_003900	c.C1175T	p.P392L	9,00x10^–4^	rs104893941	1,5x10^–4^	2,20x10^–3^	Likely pathogenic	D/D/D/D/B/A/L/0.996	2	Fecto et al., 2011	#57u, #64u
*SQSTM1*	NM_003900	c.G1165C	p.E389Q	0	rs1391182750	0	0	VUS	D/D/N/T/B/D/L/0.99	1	–	#73u
*SQSTM1*	NM_003900	c.G1178A	p.R393Q	4,94x10^–5^	rs200551825	0	4,24x10^–5^	Likely pathogenic	D/D/N/D/P/D/M/0.999	1	Kwok et al., 2014	#108u
*TBK1*	NM_013254	c.1888_1890del	p.K631del	0	–	0	0	VUS	–	1	–	#90u
*TBK1*	NM_013254	c.T1190C	p.I397T	1,00x10^–4^	rs755069538	0	0	Likely pathogenic	T/T/N/T/B/D/L/0.908	1	Pozzi et al., 2017	#97u
*UBQLN2*	NM_013444	c.A1174G	p.M392V	0	rs1384003425	0	0	Likely pathogenic	T/T/N/T/B/D/L/0.955	1	Huang et al., 2017	#91u
*UBQLN2*	NM_013444	c.A252T	p.Q84H	0	–	0	0	VUS	T/T/N/T/D/D/L/0.871	1	–	#111u

PopMax MAF (ExAc), Maximal general minor allele frequency of the variant in the ExAc database; ACMG, guideline of the American College of Medical Genetics and Genomics; dbSNP, Single Nucleotide Polymorphism Database reference SNP ID number for the variant; ALSdb MAF ALS, Minor allele frequency in ALS Data Browser (ALSdb) variants from 3,239 ALS cases and 11,808 con; ALSdb MAF Control, Minor allele frequency in ALS Data Browser (ALSdb) containing variants from 11,808 controls; No patients, Number of patients with this variant in this study; VUS, variant of uncertain significance; MetaSVM and MetaLR prediction: D, Damaging, T, Tolerated; PROVEAN: D, Deleterious, N, Neutral; SIFT: D, Deleterious, T, Tolerated; PolyPhen2: D, Damaging, B, Benign; MutTast (Mutation Taster): D, Disease causing, A, Disease causing automatic, MutAs (Mutation Assessor): N, Neutral; L, Low; M, Medium; H, High; DANN, The value range is 0 to 1, with 1 given to the variants predicted to be the most damaging.

Six (6/107, 5.61%) patients had two rare variants in different major ALS genes. Two of these patients carried the C9orf72 RE and additional variants in the SQSTM1 or NEK1 genes. Only one patient (#108u) was detected to carry a pathogenic and a likely pathogenic variant in two different major ALS genes. In case of the other five patients with multiple major ALS gene variants, at least one of the two variants was categorized as VUS (**Table 3**).

**Table 3:** Patients with two major ALS gene variants.

Patient ID	Age of onset (age range group, years)	Duration before the 1^st^ exam	Early signs and symptoms	Symptoms	Gene	Variant	PopMax MAF (ExAc)	dbSNP	Pathogenicity (ACMG)	Other disease
#73u	65-70	1.5 y	Tetraparesis	B, PB ++, LMN ++, UMN +++	*SQSTM1*	p.E389Q	0	rs1391182750	VUS	Paget disease, Hyperparathyreodism, Hypothyreodism, Colon cancer (operated)
					*SIGMAR1*	p.I42R	0	rs1206984068	VUS	
#85u	60-65	6 months	Four extremity weakness with spasticity with muscle atrophy and fasciculations, especially in the interosseus muscles	B, PB +, LMN ++, UMN ++	*CCNF*	p.L106V	0	rs990719669	VUS	
					*KIF5A*	p.E758K	5.54×10^–4^	rs140281678	VUS	
#90u	35-40	6 months	Psychomotor activity was slowing down, corticospinal tract lesion signs bilaterally	Memory loss, dementia.B, PB+, LMN ++, UMN +++	*TBK1*	p.K631del	3.73×10^–3^	–	VUS	–
					*NEK1*	p.R261H		rs200161	VUS	
#99u	55-60	8 months	Dysarthria	B, PB +++, LMN ++, UMN+	*NEK1*	p.N250S	0	rs368762503	VUS	Hypothyreoidism
					*C9orf72*	Repeat expansion	0	–	Pathogenic	
#106u	50-55	9 months	Four extremity weakness with spasticity	B, PB –, UMN+++, LMN+++	*GRN*	p.C335R	0	–	VUS	Alcoholism, polyneuropathy, cervical myelopathy caused by CIII-IV-V. disc protrusions
					*NEFH*	p.P505L	0	rs1414968372	VUS	
#108u	45-50	2 years	Dysarthria	UMN, B, PB+++	*SQSTM1*	p.R393Q		rs200551825	VUS	–
				LMN+++, UMN+++, B	*C9orf72*	Repeat expansion	0	–	Pathogenic	

B, PB, Bulbar and pseudobulbar, UMN, upper motor neuron, LMN, lower motor neuron, PopMax MAF (ExAc), Maximal general minor allele frequency of the variant in the ExAc database; ACMG, guideline of the American College of Medical Genetics and Genomics; dbSNP, Single Nucleotide Polymorphism Database reference SNP ID number for the variant; VUS, variant of uncertain significance.

Additionally, a novel variant (c.-25C > T) in the 5′ untranslated region of the *FUS* gene was also detected. As the screening of untranslated regions was not in the scope of our research, we did not examine it further.

No SOD1 and TARDBP gene variants were found in this cohort. We would like to point out that 37 of the analyzed samples were overlapping samples from a previous study (Tripolszki et al., 2017a) and were known to be negative for SOD1 and TARDBP mutations. Still, based on earlier results, one would expect to detect SOD1 variants in the further 70 samples.

### Variants Detected in Minor ALS Genes

By focusing on the analysis of minor ALS genes, 33 variants (31 missense and 2 splicing) were detected in 26 genes corresponding to 29 patients (27.1% of all patients, **Supplementary Table 2**). No patients were identified as being homozygous for any of the detected variants.

A patient was carrying two novel variants (T2583I and G4290R) in the *DYNC1H1* gene; both variants localized in the motor domain of the protein. Due to the limitation of short-read sequencing and the lack of parental DNA, we could not assess whether the two variants were present in a compound heterozygous state or as a complex allele. Several other candidate variants of uncertain significance were identified (**Supplementary Table 2**) in minor genes. Results of future replication studies will reveal which of these variants are truly causative.

### Genetic Variants of Genes Related to Other Neurodegenerative and Neuromuscular Diseases

Additionally, an analysis of 116 genes of other neurodegenerative diseases was performed to reveal potentially disease-causing variants. A total of 48 missense variants in 31 different genes were identified in 41 of our patients. Two of these variants were classified as likely pathogenic and 46 as VUS (**Supplementary Table 3**).

Among others, a known missense variant was detected in the *GJB1* gene (R230C), which is associated with mitochondrial disorders and Charcot–Marie–Tooth disease, and two variants of conflicting significance in the *GBE1* gene (H398R and R166C), which is associated with autosomal recessive adult-type polyglucosan body disease (Online Mendelian Inheritance in Man). We identified sequence alterations in additional genes that are listed in **Supplementary Table 3**; however, most of these variants are unlikely to be implicated in our patients’ phenotypes.

## Discussion

The frequency of causative variants in ALS patients has been extensively investigated in populations of different ethnic origins. Here, we used a combination of repeat-sizing of the *C9orf72* gene and next-generation sequencing to perform a comprehensive genetic analysis of 107 Hungarian ALS patients. Our genetic analysis included all known ALS-associated major and minor genes and an additional list of genes associated with other neurogenetic diseases.

Including the *C9orf72* RE, a total of 29 genetic variations have been detected in 14 different major ALS genes, leading to a positive result in 36.45% (39/107) of our ALS patients (**Table 2**). According to ACMG variant classification, 2 of the 29 detected major ALS variants were categorized as pathogenic, 4 as likely pathogenic and the remaining 23 variants as variants of uncertain significance. Furthermore, 33 variants in 26 minor ALS genes (**Supplementary Table 2**) and 48 variants in 31 genes associated with other neurodegenerative diseases (**Supplementary Table 3**) were detected. A major challenge of using NGS data is the critical evaluation of the significance of detected variants, especially those that are very rare or novel. While the disease-causing role of several variants identified in this study has previously been well established (ALSoD, Abel et al., 2012), other variants may show reduced penetrance or may be rare benign alterations.

In accordance with the previous cohort studies, the most frequent genetic alteration was the *C9orf72* repeat expansion, detected in 10 patients (9.3%) of this cohort. The initial signs of ALS in these patients were predominantly bulbar dysfunctions, especially altered speech (seven out of 10 patients). It is known that *C9orf72* repeat expansion has been found in ∼7% of sporadic ALS cases of European ancestry (Renton et al., 2014). Several studies reported missense variants in the coding region of the *C9orf72* gene (Kenna et al., 2013; Koppers et al., 2013; Krüger et al., 2016), but the relevance of *C9orf72* variants detected in the coding region is not yet understood. Therefore, we cannot determine the importance of the R431Q missense variant detected in this study.

We identified two missense variants in the *NEK1* gene (both classified as VUS): the R261H variant in five and the N250S variant in a single patient. The *NEK1* gene has been recently recategorized as a major ALS-associated gene (Kenna et al., 2016), following two prior studies that identified *NEK1* as an ALS candidate gene (Cirulli et al., 2015; Brenner et al., 2016). R261H was earlier described to significantly increase ALS risk in both fALS and sALS in independent cohort studies (Brenner et al., 2016; Kenna et al., 2016; Gratten et al., 2017; Nguyen et al., 2018). In our evaluation, we observed an enrichment of the R261H variant in patients [5/107 (4.67%), MAF = 0.0234 patients vs controls 4/370 (1.08%), MAF = 0.0054]. According to the gnomAD and Kaviar databases, the maximum allele frequency of the R261H variant is 0.004 (Glusman et al., 2011;Lek et al., 2016). Earlier studies detected R261H in 1.8% of ALS patients and 0.66% of controls with minor allele frequencies 0.009 and 0.0033, respectively (Nguyen et al., 2017). Based on these results, we assume that the *NEK1* R261H variant is more frequent in the Hungarian population (both in patients and controls) than in other populations, although further large cohort studies are needed to confirm this conclusion. This study provides additional evidence that *NEK1* missense variants may contribute to the development of sALS.

Missense variants in the *NEFH* gene were detected in four patients: the T338I variant in two cases and the R148P and P505L variants in single cases. *NEFH* encodes the heavy neurofilament protein, and its variants have been associated with neuronal damage in ALS (Figlewicz et al., 1994). The T338I and R148P variants affect the conserved central coiled-coil rod domain of the protein mediating dimerization; therefore, we suggest their potential deleterious effect on the protein. In the individual carrying the P505L *NEFH* variant, an additional novel alteration (C335R) was detected in the *GRN* gene. Loss-of-function *GRN* variants are primarily considered to cause frontotemporal lobar degeneration (Mackenzie et al., 2006), but there is evidence that missense *GRN* variants are also linked to the pathogenesis of ALS (Sleegers et al., 2008). The novel *GRN* variant reported in this study results in a cysteine-to-arginine change in the cysteine-rich granulin A domain.

Four cases were identified to carry *SQSTM1* variants: the P392L in two cases and the E389Q and R393Q in single patients. All three alterations are located within the C-terminal ubiquitin-associated (UBA) end of the sequestome 1 protein. Variants of the *SQSTM1* gene were originally reported in Paget’s disease of bone (Laurin et al., 2002). However, recent publications suggest a link between *SQSTM1* variants and ALS/FTD (Fecto et al., 2011). The P392L and R393Q variants are known variants reported by other study groups (Fecto et al., 2011; Kwok et al., 2014). Interestingly, the patient (#73u) carrying the novel E389Q variant was also diagnosed with Paget’s disease of bone. In addition, this patient also carried a variant of unknown significance (I42R) in the *SIGMAR1* gene in heterozygous form. This case exemplifies the relevant observation of phenotypic pleiotropy and highlights the complexity of the phenotype–genotype correlation.

Variants in the *KIF5A* gene has been previously linked to autosomal dominant hereditary spastic paraparesis (SPG10) and to Charcot–Marie–Tooth disease type 2 (CMT2; Reid et al., 2002; Crimella et al., 2016; Liu et al., 2014; Jennings et al., 2017). Nonetheless, recent studies proved that *KIF5A* variants have a role in ALS (Brenner et al., 2018; Nicolas et al., 2018). According to earlier studies, *KIF5A* variants described in SPG10 or CMT2 patients occur in the kinesin motor domain (amino acid positions 9–327) and in the alpha-helical coiled-coil domain (amino acid positions 331–906) (Kaji et al., 2016; Guinto et al., 2017). In contrast, variants causing ALS are found in the C-terminal cargo-binding domain (amino acids 907–1032). In the present study, we found two variants: the E758K variant in two patients and the A579T variant in one case, with both variants located within the coiled-coil domain (amino acid positions 331–906) of the protein, which is not in line with previous findings. Without additional functional evidence, the pathogenicity of these variants is uncertain.

Three rare missense variants (R2034Q, L2118V, and E2003D) of the *SPG11* gene were found. The high detection rate of missense variants of this gene is probably due to the large size of the coding region; therefore, we suggest that these SPG11 variants are unlikely to be deleterious. Variants in the *SPG11* gene are most commonly associated with autosomal recessive spastic paraplegia, although homozygous variants have been recently identified in juvenile ALS (Orlacchio et al., 2010; Daoud et al., 2012), and heterozygous missense variants in sALS (Kenna et al., 2013; Couthouis et al., 2014).

Variants in *UBQLN2* have been shown to be a cause of dominant X-linked ALS (Deng et al., 2011). A previously reported (M392V, Huang et al., 2017) and a novel variant (Q84H) were found in the *UBQLN2* gene. The novel Q84H variant affects the N-terminal ubiquitin-like domain of the ubiquilin-2 protein, which is involved in binding to proteasome subunits (Ko et al., 2004).

*FUS* variants have been mostly detected in familial ALS cases that are localized within the C-terminus of the FUS protein (Shang and Huang, 2016). However, the two rare *FUS* variants (Y25C and P106L) that were detected in this study were located in the N-terminal “prion-like” Q/G/S/Y domain (amino acids 1–165) of the protein. Although the majority of FUS mutations linked to ALS are located in the extreme C-terminus of the protein, several studies show that N-terminal variants may also be damaging (Nomura et al., 2014, Murakami et al., 2015).

In the TBK1 gene, a known missense variant (I397T) and a novel non-frameshift deletion (K631del) were identified in our patient cohort. The patient (#90u) carrying the novel K631del deletion was a 37-year-old patient who also showed symptoms of frontotemporal dementia (FTD). This is in line with the data from previous studies; according to which, *TBK1* is a causative gene of ALS–FTD (Cirulli et al., 2015, Freischmidt et al., 2015). The *NEK1* R261H variant was also present in this patient. A combined effect of the two major ALS gene variants may contribute to the early onset and fast progression of the disease in patient #90.

*CCNF* variants are a rare cause of ALS–FTD; in diverse geographic familial cohorts, variants in *CCNF* were present at frequencies ranging from 0.6 to 3.3% (Williams et al., 2016). In this Hungarian cohort, we identified two patients (1.9%) with *CCNF* variants (L106V and R572W). The detected R572W variant affects the nuclear localization signal 2 (amino acids 568–574) of the CCNF protein.

A previously characterized pathogenic nonsense variant (G1177X) and a rare missense alteration (R1499H) were detected in the *ALS2* gene, both in heterozygous form. The alsin protein encoded by the *ALS2* gene is involved in endosome/membrane trafficking and fusion, cytoskeletal organization, and neuronal development/maintenance (Hadano et al., 2007). Both homozygous and compound heterozygous variants in the *ALS2* gene have been described as causative for juvenile ALS (Yang et al., 2001). The G1177X nonsense variant was first detected in compound heterozygous form in a family with two affected siblings suffering from infantile ascending spastic paralysis with bulbar involvement (Sztriha et al., 2008). The ages of onset of the patients with the *ALS2* variants reported in this study were later than juvenile ALS onset, which generally manifests before 25 years of age (Orban et al., 2007). Previous studies suggested that heterozygous variants in the *ALS2* may be causative for adult-onset sALS (Kenna et al., 2013; Couthouis et al., 2014).

*MATR3* encodes three protein isoforms that have been described as nuclear-matrix and DNA/RNA binding proteins involved in transcription and stabilization of mRNA (Belgrader et al., 1991; Salton et al., 2011; Coelho et al., 2015). In the present study, two novel heterozygous variants (P11S, S275N) were detected. The P11S variant affects the b isoform of the MATR3 protein (NM_001194956 and NP_001181885), contributing to splicing alteration of other isoforms. Further evidence is required to elucidate the mechanism of pathogenicity of these alterations.

We discovered several variants in ALS candidate and risk genes. In a patient with LMN-dominant ALS with slow progression, we found two novel variants (T2583I and G4290R) in the *DYNC1H1* gene. Variants in the *DYNC1H1* gene result in impairment of retrograde axonal transport leading to progressive motor neuron degeneration in mice (Hafezparast et al., 2003) and have been described in a range of neurogenetic diseases, including Charcot–Marie–Tooth type 2O, spinal muscular atrophy, and hereditary spastic paraplegia (Weedon et al., 2011; Harms et al., 2012; Poirier et al., 2013; Strickland et al., 2015; Beecroft et al., 2017). A few studies described heterozygous variants in the *DYNC1H1* gene in fALS and sALS patients, suggesting its role in ALS (Puls et al., 2003; Münch et al., 2004). Based on our findings, we strengthen the potential link between *DYNC1H1* variants and ALS.

Given that there are genetic and symptomatic overlaps among many neurodegenerative diseases, it has been suggested that causative variants might play roles in multiple disorders (Pang et al., 2017). Two heterozygous variants (H398R and R166C) were detected in the *GBE1* gene. This gene is associated with autosomal recessive adult polyglucosan body disease (APBD), which is characterized by UMN signs, cognitive impairment, and decreased activity of the glycogen branching enzyme (Lossos et al., 1998). *GBE1* variants have been recently detected in German ALS patients (Krüger et al., 2016). Although the majority of *GBE1 *disease-causing variants were detected in homozygous or compound heterozygous form, a substantial percentage of individuals with APBD carry a single variant in one allele (Ubogu et al., 2005; Akman et al., 2015).

An oligogenic model of ALS has been proposed (van Blitterswijk et al., 2012), with several studies suggesting that ALS may be caused by a single highly penetrant variant or a combination of several less penetrant variants (Martin et al., 2017). In addition, environmental factors have also been implicated in disease development (Fang et al., 2009). In earlier studies, the frequency of patients with more than one major ALS gene variants was ranging from 1.6% to 3.8% (Kenna et al., 2013; Cady et al., 2015; Zhang et al., 2018). In this study, we describe six patients (6/107, 5.61%) with two variants in major ALS genes (**Table 3**). Only patient #108u was detected to carry a pathogenic and a likely pathogenic variant in two different major ALS genes; in case of the other five patients with multiple major ALS gene variants, at least one of the two variants was categorized as VUS (**Table 3**). Co-occurrence of multiple variants is most frequently observed in patients who carry the C9orf72 RE (Nguyen et al., 2018). Two of the six patients with multiple variants in our cohort carried the *C9orf72* RE and additional variants in the *SQSTM1* or *NEK1* genes. In addition, it has been described that oligogenic inheritance is also associated with an earlier age of onset and rapid disease progression (Cady et al., 2015; Nguyen et al., 2018). In our cohort, most of the patients with two variants showed earlier onset, faster progression, or both, although a cohort of larger size is needed to confirm these observations. Additionally, many of our cases with major ALS gene variants also have several variants in other risk genes (**Supplementary Table 2**) or in genes associated with other diseases (**Supplementary Table 3**); still, the relevance of these results will only become clear when additional larger cohorts are studied.

Our results support the hypothesis that sALS has a complex model of inheritance, in which multiple variants and environmental factors contribute to disease susceptibility (van Blitterswijk et al., 2012; Martin et al., 2017). In general, this cohort of 107 ALS cases uncovers a heterogeneous genetic architecture with variants in numerous major and minor ALS genes. Several major ALS genes have been also linked to other diseases such as *SQSTM1*—Paget disease, and *KIF5A*—spastic paraplegia 10. In line with this, our results support the observation of phenotypic pleiotropy, where variants of a single gene contribute to different phenotypes. These findings further highlight the necessity for large-scale multicenter studies on ALS patients for a better understanding of the underlying genetic causes. Large-scale consortium approaches, such as Project MinE, will improve the separation of true causative genetic variants from irrelevant ones, which will help to gain a more accurate view of the genetic pattern of ALS. With this study, which represents the first comprehensive genetic study in the Hungarian ALS patients, we contribute to this approach.

## Data Availability

The raw sequencing data of the 107 patients have been deposited in the NCBI Sequence Read Archive with BioProject accession no. PRJNA549957 (https://www.ncbi.nlm.nih.gov/sra/PRJNA549957).

## Ethics Statement

The investigation was approved by the Hungarian Investigational Review Board at University of Szeged and the Ethics Committee at Medical University of Graz. Written informed consent was obtained from patients and healthy individuals, and the study was conducted according to the principles of the Declaration of Helsinki.

## Author Contributions

Conception, design, and coordination of the study were performed by KT, JE, DN, and MS. Acquisition of clinical data and sample collection were performed by JE, PK, PG, and HS. Analysis and interpretation of data were done by KT, JE, PG, ZN, and DN. Drafting of the manuscript was done by KT, JE, and ZN. Revision of the manuscript was done by KT, MS, PG, JE, PK, HS, and DN.

## Funding

This work was funded by the Hungarian Brain Research Program (grant no. 2017-1.2.1-NKP-2017-00002), and the GSHA is a part of an EU Joint Programme—Neurodegenerative Disease Research (JPND) project. The project is supported in Austria by the Austrian Science Fund under the aegis of JPND—www.jpnd.eu. This work was conducted with the support of the Szeged Scientists Academy under the sponsorship of the Hungarian Ministry of Human Capacities (EMMI: 13725-2/2018/INTFIN).

## Conflict of Interest Statement

The authors declare that the research was conducted in the absence of any commercial or financial relationships that could be construed as a potential conflict of interest.

The reviewer SK declared a past co-authorship with one of the authors MS to the handling editor.
