# Supplementary material for: Comprehensive Genetic Analysis of a Hungarian Amyotrophic Lateral Sclerosis Cohort
Source: Front Genet. 2019 Aug 16;10:732. doi: 10.3389/fgene.2019.00732 (PMC6707335; doi:10.3389/fgene.2019.00732)
Supplement: Supplementary file 1 [file DataSheet_1.pdf]

Supplementary Table 1. Gene lists used in this study

Gene set 1 (n=30)	Gene set 2 (n=101)	Gene set 3 (n=116)	
Major ALS genes	Minor (risk or candidate) genes	Other disease genes	Disease names linked to Gene set 3 - based on OMIM terminology
ALS2	*C21orf2*	*AARS*	Charcot-Marie-Tooth disease, axonal, type 2N
ATXN2	*EPB41L1*	*ACTA1*	Myopathy, scapulohumeroperoneal, Myopathy, actin, congenital, with cores, Myopathy, actin, congenital, with excess of thin myofilaments, Myopathy, congenital, with fiber-type disproportion 1, Nemaline myopathy 3, autosomal dominant or recessive
C9orf72	*EPHA4*	*AFG3L2*	Spinocerebellar ataxia 28
CCNF	*HNRNPA2B1*	*ALDH18A1*	Spastic paraplegia 9A, autosomal dominant, Spastic paraplegia 9B, autosomal recessive
CHCHD10	*LMNB1*	*ANO5*	Spastic paraplegia 9A, autosomal dominant, Spastic paraplegia 9B, autosomal recessive
CHMP2B	*MAPT*	*AP4M1*	Spastic paraplegia 50, autosomal recessive
DAO	*MOBP*	*APTX*	Ataxia-oculomotor apraxia syndrome
DCTN1	*PON1*	*ATG5*	Spinocerebellar ataxia, autosomal recessive 25
KIF5A	*PON2*	*ATL1*	Neuropathy, hereditary sensory, type ID, Spastic paraplegia 3A, autosomal dominant
FUS	*PON3*	*ATM*	Ataxia-telangiectasia
GLE1	*PRPH*	*BAG3*	Myopathy, myofibrillar, 6
GRN	*PSEN1*	*BICD2*	Spinal muscular atrophy, lower extremity-predominant, 2, AD
HNRNPA1	*SARM1*	*BSCL2*	Spastic paraplegia type 17, Distal hereditary motor neuropathy type 5A
MATR3	*SCFD1*	*C10orf2*	Progressive external ophthalmoplegia with mitochondrial DNA deletions, autosomal dominant 3
NEFH	*SPG7*	*CA8*	Cerebellar ataxia and mental retardation with or without quadrupedal locomotion 3
NEK1	*TREM2*	*CACNA1A*	Spinocerebellar ataxia 6
OPTN	*UNC13A*	*CCDC88C*	Spinocerebellar ataxia 40
PFN1	*VEGFA*	*CLN5*	Ceroid lipofuscinosis, neuronal, 5
SIGMAR1	*AGT*	*CNBP*	Myotonic dystrophy 2
SOD1	*ALAD*	*COX20*	Mitochondrial complex IV deficiency
SPG11	*APEX1*	*CPT1C*	Spastic paraplegia 73, autosomal dominant
SQSTM1	*APOE*	*CRYAB*	Myopathy, myofibrillar, 2
SS18L1	*AR*	*DES*	Muscular dystrophy, limb-girdle, type 2R, Myopathy, myofibrillar, 1
TAF15	*ARHGEF28*	*DHTKD1*	Charcot-Marie-Tooth disease, axonal, type 2Q
TARDBP	*B4GALT6*	*DMPK*	Myotonic dystrophy 1
TBK1	*BCL11B*	*DNAJB2*	Spinal muscular atrophy, distal, autosomal recessive, 5
TUBA4A	*BCL6*	*DNM2*	Charcot-Marie-Tooth disease, axonal type 2M
UBQLN2	*C1orf27*	*DRP2*	Charcot-Marie-Tooth neuropathy, X-linked dominant, 1 candidate gene
VAPB	*CCS*	*EEF2*	Spinocerebellar ataxia 26
VCP	*CDH13*	*EGR2*	Charcot-Marie-Tooth disease, type 1D
	CDH22	*ELOVL4*	Spinocerebellar ataxia 34
	CHGB	*ELOVL5*	Spinocerebellar ataxia 38
	CNTF	*ERLIN1*	Spastic paraplegia 62
	CNTN4	*FBLN5*	Neuropathy, hereditary, with or without age-related macular degeneration
	CNTN6	*FGD4*	Charcot-Marie-Tooth disease, type 4H
	CRIM1	*FGF14*	Spinocerebellar ataxia 27
	CRYM	*FLNC*	Myofibrillar, distal myopathy
	CSNK1G3	*GDAP1*	Charcot-Marie-Tooth disease, axonal, type 2K, Charcot-Marie-Tooth disease, type 4A
	CST3	*GJB1*	Charcot-Marie-Tooth disease type X1
	CX3CR1	*GNB4*	Charcot-Marie-Tooth disease, dominant intermediate F
	CYP2D6	*GNE*	Nonaka myopathy
	DIAPH3	*GRID2*	Spinocerebellar ataxia, autosomal recessive 18
	DISC1	*HADHB*	Trifunctional protein deficiency
	DOC2B	*HSPB1*	Charcot-Marie-Tooth disease, axonal, type 2F/ Neuropathy, distal hereditary motor, type IIB
	DPP6	*HSPB8*	Charcot-Marie-Tooth disease, axonal, type 2L/ Neuropathy, distal hereditary motor, type IIA
	DYNC1H1	*HSPD1*	Spastic paraplegia 13, autosomal dominant
	EFEMP1	*IGHMBP2*	Charcot-Marie-Tooth disease type2S
	FEZF2	*INF2*	Charcot-Marie-Tooth disease type dominant intermediate E
	FGGY	*ITPR1*	Spinocerebellar ataxia 15/Spinocerebellar ataxia 29, congenital nonprogressive
	GARS	*KBTBD13*	Nemaline myopathy 6, autosomal dominant
	GRB14	*KCNA2*	Early infantile epileptic encephalopathy
	HEXA	*KCND3*	Spinocerebellar ataxia 19
	HFE	*KIAA0196*	Spastic paraplegia 8, autosomal dominant
	ITPR2	*KIF1B*	Charcot-Marie-Tooth disease type 2A1
	KDR	*LDB3*	Myopathy, myofibrillar, 4
	KIFAP3	*LITAF*	Charcot-Marie-Tooth disease, type 1C
	LIF	*LMNA*	Limb-girdle muscular dystrophy type 1B
	LIPC	*MARS*	Charcot-Marie-Tooth disease, axonal, type 2U
	LOX	*MED25*	Charcot-Marie-Tooth disease type 2B2
	LUM	*MFN2*	Charcot-Marie-Tooth disease, axonal, type 2A2A/ Hereditary motor and sensory neuropathy VIA
	MAOB	*MORC2*	Charcot-Marie-Tooth disease, axonal, type 2Z
	MT-ND2	*MPZ*	Charcot-Marie-Tooth disease, dominant intermediate D / Charcot-Marie-Tooth disease, type 1B/ Charcot-Marie-Tooth disease, type 2I/ Neuropathy, congenital hypomyelinating
	NAIP	*MRE11A*	Ataxia-telangiectasia-like disorder 1
	NETO1	*MTMR2*	Charcot-Marie-Tooth disease, type 4B1
	NIPA1	*MYH7*	Laing distal myopathy/ Myopathy, myosin storage, autosomal dominant
	NT5C1A	*MYOT*	Muscular dystrophy, limb-girdle, type 1A
	OGG1	*NDRG1*	Charcot-Marie-Tooth disease type 4D
	OMA1	*NEFL*	Charcot-Marie-Tooth disease, dominant intermediate G/ Charcot-Marie-Tooth disease, type 1F, 2E
	PARK7	*NKX2-1*	Chorea, hereditary benign
	PCP4	*NPC1*	Niemann-Pick disease, type C1, D
	PLEKHG5	*NPC2*	Niemann-pick disease, type C2
	PVR	*PCNA*	Ataxia-telangiectasia-like disorder 2
	RAMP3	*PDYN*	Spinocerebellar ataxia 23
	RBMS1	*PEX10*	Peroxisome biogenesis disorder 6A, 6B
	RNASE2	*PIK3R5*	Ataxia-oculomotor apraxia 3
	RNF19A	*PLP1*	Spastic paraplegia 2, X-linked
	SCN7A	*PMP22*	Neuropathy, inflammatory demyelinating, Charcot-Marie-Tooth disease, type 1A, 1E
	SELL	*PNKP*	Ataxia-oculomotor apraxia 4
	SEMA6A	*PNPLA2*	Neutral lipid storage disease with myopathy
	SLC1A2	*POLG*	Progressive external ophthalmoplegia type A1, Sensory ataxic neuropathy dysarthria and ophthalmoparesis
	SLC39A11	*PRKCG*	Spinocerebellar ataxia 14
	SMN1	*PRPS1*	Charcot-Marie-Tooth disease, X-linked recessive, 5
	SMN2	*PRX*	Charcot-Marie-Tooth disease type 4F
	SNCG	*RAB7A*	Charcot-Marie-Tooth disease type 2B
	SOD2	*REEP1*	Neuronopathy, distal hereditary motor, type VB, Spastic paraplegia 31
	SOX5	*REEP2*	Spastic paraplegia 72
	SUSD1	*RTN2*	Spastic paraplegia 12
	SYNE1	*SACS*	Spastic ataxia type Charlevoix-Saguenay
	SYT9	*SBF1*	Charcot-Marie-Tooth disease type 4B3
	TRPM7	*SBF2*	Charcot-Marie-Tooth disease, type 4B2
	VDR	*SEPT9*	Amyotrophy, hereditary neuralgic
	VPS54	*SH3TC2*	Mononeuropathy of the median nerve, Charcot-Marie-Tooth disease type 4C
	ZFP64	*SIL1*	Marinesco-Sjogren syndrome
	ZNF512B	*SLC33A1*	Spastic paraplegia 42
	ZNF746	*SOX10*	PCWH syndrome
	EWSR1	*SPAST*	Spastic paraplegia 4, autosomal dominant
	ANG	*SPTBN2*	Spinocerebellar ataxia 5
	ELP3	*STUB1*	Spinocerebellar ataxia type 16
	ERBB4	*TFG*	Hereditary motor and sensory neuropathy, Okinawa type/ ?Spastic paraplegia 57, autosomal recessive
	FIG4	*TGM6*	Spinocerebellar ataxia 35
	SETX	*TIA1*	Welander distal myopathy
		TMEM240	Spinocerebellar ataxia 21
		TPM2	Nemaline or cap myopathy
		TPM3	CAP myopathy 1/ Myopathy, congenital, with fiber-type disproportion/ Nemaline myopathy 1
		TPP1	Spinocerebellar ataxia, autosomal recessive 7/ Ceroid lipofuscinosis, neuronal, 2
		TRIM2	Charcot-Marie-Tooth disease, type 2R
		TRPC3	Spinocerebellar ataxia type 41
		TRPV4	Hereditary motor and sensory neuropathy, type Iic/ Scapuloperoneal spinal muscular atrophy/ Spinal muscular atrophy, distal, congenital nonprogressive
		TSEN54	Pontocerebellar hypoplasia type 5, 2A, 4
		TTBK2	Spinocerebellar ataxia 11
		TTPA	Ataxia with isolated vitamin E deficiency
		YARS	Charcot-Marie-Tooth disease, dominant intermediate C
		ZFYVE26	Spastic paraplegia type 15
		GBE1	Polyglucosan body neuropathy, adult form (APBN)
		LRSAM1	Charcot-Marie-Tooth disease type 2P
		WASHC5	Spastic paraplegia 8, autosomal dominant

Supplementary Table 2. Variants detected in minor ALS genes in this study

Patient ID	Gene	Transcript ID	Nucleotide change	Amino acid change	Variant description	PopMax MAF (ExAc)	ALSdb MAF ALS	ALSdb MAF Control	dbSNP	Pathogenicity (ACMG)
#48r	*CCS*	NM_005125	c.490-1G>A	-	splicing	0	0	0	rs1199737977	VUS
#2u	*CDH13*	NM_001220488	c.A713G	p.N238S	nonsyn SNV	0	3,09X10^{-4}	0.00008469	rs757294994	VUS
#59r	*CDH22*	NM_021248	c.G352A	p.D118N	nonsyn SNV	1,65X10^{-5}	0	0	rs372007812	VUS
#84u	*CNTN6*	NM_014461	c.G2222C	p.R741P	nonsyn SNV	0	0	0	rs754797179	VUS
#56r	*DIAPH3*	NM_001042517	c.G1445A	p.R482Q	nonsyn SNV	0	0	0	rs762394580	VUS
#80r	*DIAPH3*	NM_001042517	c.C3554G	p.A1185G	nonsyn SNV	0	0	0	-	VUS
#64r	*DISC1*	NM_001012958	c.C1055G	p.P352R	nonsyn SNV	0	0	0	rs750722558	VUS
#113u	*DISC1*	NM_001164550	c.T1163C	p.L388P	nonsyn SNV	0	0	0	rs1196077554	VUS
#80u	*DPP6*	NM_130797	c.A1424G	p.D475G	nonsyn SNV	0	0	0	-	VUS
#54r	*DYNC1H1*	NM_001376	c.C7748T	p.T2583I	nonsyn SNV	0	0	0	-	VUS
#54r	*DYNC1H1*	NM_001376	c.G12868C	p.G4290R	nonsyn SNV	0	0	0	-	VUS
#87u	*DYNC1H1*	NM_001376	c.C11221T	p.R3741C	nonsyn SNV	0	0	0	-	VUS
#50u	*EPB41L1*	NM_012156	c.C1123T	p.R375W	nonsyn SNV	0	0	0	rs758635781	VUS
#69u	*EPHA4*	NM_004438	c.C281T	p.A94V	nonsyn SNV	0	0	0	rs1347356714	VUS
#71u	*ERBB4*	NM_005235	c.G268T	p.A90S	nonsyn SNV	5,14X10^{-5}	0	8,47X10^{-5}	rs201678258	VUS
#97u	*FEZF2*	NM_018008	c.G675T	p.K225N	nonsyn SNV	0	0	0	-	VUS
#94u	*FIG4*	NM_014845	c.C2095T	p.R699C	nonsyn SNV	0	0	8,47X10^{-5}	rs764799053	VUS
#55u	*GRB14*	NM_004490	c.C1007T	p.A336V	nonsyn SNV	0	0	0	rs756507556	VUS
#114u	*GRB14*	NM_004490	c.C386T	p.T129M	nonsyn SNV	5,78X10^{-5}	0	4,23X10^{-5}	rs141568578	VUS
#82r	*HEXA*	NM_000520	c.1073+1G>C	-	splicing	2,25X10^{-4}	4,64X10^{-4}	6,80X10^{-4}	rs76173977	Likely pathogenic
#88u	*KDR*	NM_002253	c.C1595T	p.A532V	nonsyn SNV	9,89X10^{-5}	0	1,27X10^{-4}	rs147066083	VUS
#100u	*KIFAP3*	NM_014970	c.A1043G	p.K348R	nonsyn SNV	0	0	0	rs1203689157	VUS
#79u	*OMA1*	NM_145243	c.T623G	p.V208G	nonsyn SNV	2,47X10^{-5}	0	0	rs552327271	VUS
#80u	*PLEKHG5*	NM_001265593	c.A1244C	p.H415P	nonsyn SNV	0	0	0	-	VUS
#111u	*PLEKHG5*	NM_001265593	c.C980T	p.P327L	nonsyn SNV	0	0	0	rs910474236	VUS
#84u	*PON2*	NM_000305	c.T705G	p.Y235X	nonsyn SNV	0	0	0	-	VUS
#75u	*PON3*	NM_000940	c.A916T	p.I306F	nonsyn SNV	5,77X10^{-5}	1,56X10^{-4}	1,69X10^{-4}	rs201661676	VUS
#57r	*SCFD1*	NM_016106	c.C608T	p.T203I	nonsyn SNV	0	0	0	rs1459319965	VUS
#57r	*SCN7A*	NM_002976	c.A1585G	p.M529V	nonsyn SNV	0	0	0	rs1462705963	VUS
#81r	*SPG7*	NM_003119	c.C1235T	p.A412V	nonsyn SNV	0	0	0	rs746668495	VUS
#122u	*SPG7*	NM_003119	c.G1813C	p.G605R	nonsyn SNV	0	0	0	-	VUS
#81u	*SUSD1*	NM_022486	c.C1135T	p.R379C	nonsyn SNV	0	0	8,47X10^{-5}	rs778163116	VUS
#91u	*VPS54*	NM_016516	c.A1078G	p.T360A	nonsyn SNV	2,62X10^{-5}	1,56X10^{-4}	8,47X10^{-5}	rs200574098	VUS

PopMax MAF (ExAc): Maximal general minor allele frequency of the variant in the ExAc database; ACMG: guideline of the American College of Medical Genetics and Genomics; dbSNP: Single Nucleotide Polymorphism Database reference SNP ID number for the variant; ALSdb MAF ALS: Minor allele frequency in ALS Data Browser (ALSdb) variants from 3,239 ALS cases; ALSdb MAF Control: Minor allele frequency in ALS Data Browser (ALSdb) containing variants from 11,808 controls; VUS: variant of uncertain significance;

Supplementary Table 3. Variants detected in other disease genes identified in ALS cases in this study

Patient ID	Gene	Transcript ID	Nucleotide change	Amino acid change	PopMax MAF (ExAc)	ALSdb MAF ALS	ALSdb MAF Control	dbSNP	Pathogenicity (ACMG)
#2u	*AFG3L2*	NM_006796	c.G499A	p.G167R	0	0	0	rs1234429070	VUS
#63u	*ALDH18A1*	NM_002860	c.G2308A	p.V770M	$2,47X10^{-5}$	0	0	rs369153920	VUS
#87u	*ATM*	NM_000051	c.A2755G	p.R919G	0	0	0	-	VUS
#102u	*ATM*	NM_000051	c.T8717C	p.V2906A	0	0	0	rs730881328	VUS
#60r	*BSCL2*	NM_001122955	c.A641G	p.H214R	$3,01X10^{-5}$	0	0	rs544020840	VUS
#64u	*CACNA1A*	NM_001127221	c.G6517T	p.D2173Y	0	0	0	rs370289732	VUS
#120u	*CACNA1A*	NM_001127221	c.G3259T	p.G1087C	0	0	0	-	VUS
#42r	*FLNC*	NM_001127487	c.C3428T	p.S1143L	0	0	0	rs756192123	VUS
#45r	*GBE1*	NM_000158	c.A1193G	p.H398R	0	0	0	rs755004170	VUS
#71r	*GBE1*	NM_000158	c.C496T	p.R166C	$1,22X10^{-4}$	0	$8,47X10^{-5}$	rs376546162	VUS
#42r	*GJB1*	NM_000166	c.C688T	p.R230C	0	$2,22X10^{-4}$	$1,73X10^{-4}$	rs587781246	Likely pathogenic
#62r	*INF2*	NM_001031714	c.G2489T	p.G830V	$1,57X10^{-5}$	$4,81X10^{-4}$	$1,72X10^{-4}$	rs377340315	VUS
#52u	*KBTBD13*	NM_001101362	c.G1112C	p.C371S	0	0	$4,48X10^{-5}$	rs1019923438	VUS
#56u	*KBTBD13*	NM_001101362	c.A239T	p.Q80L	0	0	0	-	VUS
#66u	*KBTBD13*	NM_001101362	c.C436T	p.R146C	0	0	0	-	VUS
#69u	*KBTBD13*	NM_001101362	c.C1232T	p.T411M	$3,39X10^{-4}$	0	0	rs372826347	VUS
#76u	*KBTBD13*	NM_001101362	c.T1322A	p.L441Q	0	0	$9,12X10^{-5}$	rs1005611902	VUS
#45r	*KCNA2*	NM_004974	c.T1351C	p.S451P	0	0	0	-	VUS
#60r	*KIF1B*	NM_015074	c.A2961C	p.K987N	0	0	0	-	VUS
#60r	*KIF1B*	NM_015074	c.G5270A	p.R1757Q	0	0	0	rs1010276300	VUS
#55u	*KIF1B*	NM_015074	c.G2942T	p.R981L	0	0	0	-	VUS
#105u	*KIF1B*	NM_015074	c.A1210G	p.S404G	0	0	0	-	VUS
#108u	*KIF1B*	NM_183416	c.C2707T	p.R903C	0	0	0	-	VUS
#54u	*LDB3*	NM_001171610	c.G1801A	p.V601I	0	0	0	rs727503130	VUS
#55r	*LMNA*	NM_001257374	c.G1184A	p.S395N	0	0	0	-	Likely pathogenic
#6u	*MORC2*	NM_014941	c.C1567T	p.R523C	$1,77X10^{-5}$	0	$4,24X10^{-5}$	rs548292999	VUS
#72u	*MTMR2*	NM_016156	c.A408C	p.E136D	0	0	0	-	VUS
#119u	*MTMR2*	NM_016156	c.G256C	p.D86H	0	0	0	-	VUS
#92u	*NDRG1*	NM_006096	c.C498A	p.N166K	0	0	$4,24X10^{-5}$	-	VUS
#46r	*NPC1*	NM_000271	c.A2959C	p.K987Q	0	0	0	rs969498640	VUS
#111u	*PNKP*	NM_007254	c.T287G	p.V96G	0	0	0	-	VUS
#60u,#64r	*POLG*	NM_001126131	c.G2243C	p.W748S	$8,43X10^{-4}$	$6,20X10^{-4}$	$2,97X10^{-4}$	rs113994097	VUS
#96u	*POLG*	NM_001126131	c.C1570G	p.P524A	$5,77X10^{-5}$	$1,55X10^{-4}$	0	rs577476988	VUS
#113u	*POLG*	NM_001126131	c.T391C	p.Y131H	$4,62X10^{-4}$	$7,59X10^{-4}$	$3,54X10^{-4}$	rs562847013	VUS
#69u	*SACS*	NM_014363	c.C6706T	p.P2236S	0	0	0	-	VUS
#72u	*SACS*	NM_014363	c.G6278A	p.R2093H	$6,63X10^{-5}$	0	0	rs150018812	VUS
#122u	*SACS*	NM_014363	c.T12491C	p.I4164T	0	0	0	-	VUS
#58r	*SBF1*	NM_002972	c.G3274A	p.E1092K	$1,03X10^{-4}$	0	0	rs371576175	VUS
#74u	*SBF2*	NM_030962	c.A2527G	p.M843V	0	0	0	rs1228700161	VUS
#51u	*SEPT9*	NM_001113491	c.G1405A	p.G469S	0	0	0	rs1039884847	VUS
#64u	*SH3TC2*	NM_024577	c.G1973A	p.R658H	$8,26X10^{-5}$	$3,14X10^{-4}$	$2,55X10^{-4}$	rs138040787	VUS
#51u	*SPTBN2*	NM_006946	c.G4396A	p.V1466M	0	$1,55X10^{-4}$	0	rs763193925	VUS
#119u	*SPTBN2*	NM_006946	c.C944T	p.S315L	0	0	0	rs1373892107	VUS
#48r	*TPM2*	NM_001301226	c.C356T	p.A119V	0	0	0	rs757873408	VUS
#79r	*TRPC3*	NM_001130698	c.G868A	p.A290T	0	0	$4,24X10^{-5}$	-	VUS
#66r	*TRPV4*	NM_021625	c.C1390T	p.R464C	$4,94X10^{-5}$	0	$1,27X10^{-4}$	rs373049874	VUS
#53r	*WASHC5*	NM_014846	c.T2644G	p.F882V	0	0	0	rs779756399	VUS
#59r	*ZFYVE26*	NM_015346	c.G6062A	p.R2021H	$5,77X10^{-5}$	0	$8,47X10^{-5}$	rs367758946	VUS

PopMax MAF (ExAc): Maximal general minor allele frequency of the variant in the ExAc database; ACMG: guideline of the American College of Medical Genetics and Genomics; dbSNP: Single Nucleotide Polymorphism Database reference SNP ID number for the variant; ALSdb MAF ALS: Minor allele frequency in ALS Data

Browser (ALSdb) variants from 3,239 ALS cases; ALSdb MAF Control: Minor allele frequency in ALS Data Browser (ALSdb) containing variants from 11,808 controls; VUS: variant of uncertain significance;
